# General practice nursing: who is cherishing this workforce?

**DOI:** 10.1080/17571472.2016.1220961

**Published:** 2016-08-17

**Authors:** Alison E While, Carol Webley-Brown

**Affiliations:** a Florence Nightingale Faculty of Nursing and Midwifery, King’s College London, London, UK; b Honor Oak Group Practice, London, UK

**Keywords:** General practice nurse, general practice nursing, workforce, primary care

## Abstract

The remodelling of the NHS requires a strong general practice nurse (GPN) workforce within general practice. The challenges facing general practice nursing are set within the current policy context and recent available evidence and illustrated by drawing upon the experience of a current GPN working in London. It is argued that there is a need to support the professional development of GPNs and nurture the next generation of potential GPNs if the current shortage of GPNs is to be addressed.

## Why this matters to us

General practice nurses (GPN) are integral to effective primary care but they are often neglected in discussions about the development of primary care and its workforce. There is a current shortage of GPNs in London and elsewhere and a third of current GPNs are expected to retire by 2020. The issues affecting the general practice nursing workforce are long standing and need addressing urgently so that its capacity and capabilities are maximised and the current shortfall remediated.

## Key message

The working conditions and professional development of GPNs require attention.

## Background

The world of general practice nursing is changing. This article will draw on the experiences of a general practice nurse (GPN [CWB]) gained over the last five years through working in ten different general practices within London. GPNs are an essential component of primary care [1] and without them general practitioners (GPs) would be even more stretched and stressed.[2] Whilst there is recognition that the GP is the backbone of primary care, the GPN is the unsung hero and an invisible clinician who needs cherishing with a whole system re-think.

Five Year Forward View [3] sets out the case for a re-modelling of the NHS so that it can meet the needs of an ageing population, many of whom have long term conditions. The proposed re-modelling recognises the importance of primary care as part of integrated care pathways to minimise hospital usage where possible. GPs are promised a ‘new deal’ with greater investment in primary care which includes increased training of GPs but remarkably there was no mention of GPNs despite their importance to long term condition care and Quality Outcome Framework (QOF) targets. This omission was rectified in General Practice Forward View [4] with an investment of at least £15 million to grow GPN capacity alongside the other promised investment in primary care and its workforce.

The Queen’s Nursing Institute (QNI) [5] surveyed 3405 GPNs (15% of the UK GPN workforce) and found that, as in the GP workforce, many were due to retire with 33.4% reporting that they were due to retire by 2020 and 43.1% reporting that their nursing team was deficient in both numbers and skills. Yet despite this apparent skill deficiency only 53.0% of the GPN sample reported that their employer always supported their professional development. One way of increasing workforce numbers is the offering of student placements [1] however only 27.0% of the sample reported that their practices offered pre-registration nursing placements compared to 61.5% offering medical student placements. This variation in student placement provision was within the context of a wide range of GP list sizes with 15.7% reporting that their practice list was under 5000 while 15.8% covered practices of more than 15,000 with two thirds (68.6%) of the sample delivering care to practice lists of 5000–14,999 patients. Within this context GPNs collect data all the time and are able at first hand to understand why patients use the services and enable behavioural change through their patient contacts albeit frequently at a ratio of one GPN to 3000 patients.

The QNI [5] survey also revealed a wide variety of working conditions (hours worked, hourly rates of pay, unpaid overtime, annual leave). For example, the hourly pay rates ranged from £14.60 to £22.00, 32.6% worked evening sessions and 18.5% worked at weekends with 22.8% reporting that they had two jobs. Only 35.0% considered that their salary reflected their role in their practice with 81.5% reporting that they did not receive salary increments based on their performance. Indeed, salary increments appeared to be a contentious issue with some GPNs having to resign in order to negotiate a pay increase. Two quotations summarised their current situation: ‘Practice Nurses are mostly employed by GPs who can pay us what they like’ and ‘We do not have an incremented pay scheme at work and do not know a GP surgery that does’ (p. 9).

Baird et al.’s [2] recent study of general practice has also noted an increase in workload but without a commensurate increase in GPN capacity despite the work becoming more complex and intense. They reported a 18.1% increase in GPN activity overall from their analysis of 30 million patient contacts across 177 practices; 70.0% increase in GPN telephone contacts and 17.4% increase in GPN face to face activity. Despite various attempts to manage demand in general practice, patient expectations, changing demography, new services including additions to the immunisation programme and public health campaigns, and new treatments mean that the workload of primary care has grown with GPNs becoming increasingly overstretched. Poignantly this King’s Fund report noted: ‘There is no surfeit of experienced practice nurses available to general practice’ (p. 63).

## Personal experience of a disconnected system

### GPN working conditions

Like many GPNs, CWB’s entry from hospital nursing was serendipitous. CWB was confident and competent in cardiovascular disease and health promotion with her accident and emergency background and recent experience as a Borough smoking cessation advisor but she needed additional training in diabetes, asthma and chronic obstructive pulmonary disease. [1,6] A general practice invited an application to a GPN post and indicated that the role comprised a significant long term care component with support to gain GPN competencies for cervical smears, travel health, child immunisations and other training needs. The reality of being the practice GPN was very different as there was little long term condition work and no training was offered because the practice was focused on QOF target delivery within the 15 h contract (3 h over 5 days) @ £15.00 plus 2 h unpaid overtime because ‘good time management’ was expected. Unsupported CWB moved practices and has repeatedly moved through nine other practices so that she can negotiate a pay increase with each additional core skill rising to her current pay of £21.00 per h. Sadly pressure to deliver without the necessary support or funded time appears to be common practice [5] and the lack of automatic pay reviews reflects the discretion of GPs.[1]

There is little incentive for GPN nurses to do more courses especially independent prescribing which requires practice support and time to complete the required assessments. Independent prescribing would take a GPN to the next level of working as an Advanced Nurse Practitioner (ANP) but there is no guarantee of financial reward. Currently contracts do not routinely reflect the role and responsibilities of the GPN although they should be reviewed with the post-holder as the workload changes. [7] The extent to which the working condition struggle reflects the power relations of the wider health system [8] is difficult to judge although the inferior treatment of BME staff is well known.[9,10]

### Continuing professional development

Support for continuing professional development (CPD) can be problematic with CW-B’s access to study days in the different practices varying from 0 to 4 while many of the better courses are 4–12 days in length. Further mandatory courses often compete with CPD both with their scheduling and approved absence for study/training. In 2014 the local Health Education England (South London HEE) sponsored free places on the postgraduate Train the Teachers course together with mentoring/coaching to support newly qualified nurses and student nurses in primary care but without recognising the constraints facing GPNs which included attendance at CGC mandatory and core role training before the end of March. The training included safeguarding in childhood and travel vaccinations study days, and updates in cervical cytology, diabetes, asthma, cancer, leg ulceration and mental health including Depot treatment. A GPN’s CPD may be further impeded by the October–March focus on the delivery of the QOF targets with the new national childhood immunisation schedule adding to the workload throughout the year.

In order to become a practice teacher (to support student nurse learning) in primary care (equivalent to a hospital clinical teacher which CWB was), a certified nurse teaching qualification must be completed which CWB undertook in her time. But like other CPD, because the GPs contend that as they have not asked for her to do the courses, there is no financial reward despite using her enhanced skill set at every available opportunity such as managing asthma patients. Additional constraints are the costs and scheduling of the courses. There are relevant e-learning courses and those offered through the Local Medical Committees but their cost is more than £500 which GPNs cannot afford. The timetabling of university based courses between October and March is also unhelpful. Few universities run courses over the summer months when GPNs have most availability and their course regulations are inflexible to the needs of those working in overstretched primary care where requirements for supervision of new skill development and certification of attained proficiency is challenging. The lack of accessibility to learning opportunities [1] undermines the positive impact of learning on practice and the consequent benefits to patients.[11]

### Professional isolation

Many GPNs are lone practitioners in their general practice and working part-time [1,5] adds to that isolation. While a GPN advisor may exist, their reach is limited especially if they are also employed part-time. Opportunities for clinical supervision and mutual professional support are not easily available especially if the local GP Forum is struggling due to local GPN shortages and a lack of leadership. If CCGs want to encourage the development of GPNs, they need to consider how best they can contribute to providing a nurturing environment and in what ways primary care needs to have structures in place to support the professional development of the GPN workforce.

### Variation in role and quality standards

In some general practices the GPN may undertake minor injury work or surgery such as the removal of lumps and tags following Patient Group Directives. In another general practice the GPN may develop and support Expert Patient groups while in another practice the GPN may undertake work associated with district nursing such as visiting patients with the GP and sometimes on their own if the Patient Group Directives permit lone visits. Some GPNs have an ANP qualification and forget or lose their GPN skills so that an ANP will not test a urine sample referring their patients to the GPN!

An added complexity is the power of the receptionist staff who may operate with or without the consent of GPs and may be relatives of key individuals making the organisational politics challenging for an ‘unrelated’ GPN. Indeed Hammond et al. [12] observed the varied dynamics across practices which reflect the contradictions within the receptionist role. Perhaps the time has come to state how many cervical smears can be safely taken in a 3 h session so that minimum standards can always be met. The Carter Review [13] may provide the appropriate structure for considering safe productivity in terms of a GPN ratio to patient activities within a fixed time to ensure clinical safety and practitioner wellbeing with the avoidance of undue stress and burnout.

## Conclusion

Being a GPN is a challenging role because it is complex compared to many other nursing roles based in hospitals and other institutional settings. The role exists within an often hidden and mysterious general practice hierarchy with plenty of titles to confuse patients and sometimes the GPNs themselves. The new role of nursing associate [14] will only add to the confusion. Health care assistants have been invaluable especially to a GPN who may be the only qualified nurse in the practice, but again the roles vary, some are more administrators or receptionists than carers.

The time has come for GPs and policy makers to recognise the value of GPNs and establish a system which rewards them appropriately and nurtures their development. Many nurses become GPNs having worked as established hospital nurses with a wealth of knowledge but were unable to combine childcare and other caring responsibilities with hospital shift work. At a time of workforce shortages primary care cannot afford to waste opportunities to recruit a new generation of GPNs to replace those retiring by 2020 nor can primary care afford to ‘squander’ the potential talents of the current GPN workforce. Perhaps supporting ageing GPNs to mentor and provide placements for nursing students [1,15,16] so that they become the next generation of GPNs would be a good first step.

## Disclosure statement

The authors have no conflict of interest to declare.
